# Transmission of Varicella Vaccine Virus, Japan

**DOI:** 10.3201/eid1510.090597

**Published:** 2009-10

**Authors:** Taketo Otsuka, Yasuyuki Gomi, Naoki Inoue, Makoto Uchiyama

**Affiliations:** Niigata University Graduate School of Medical and Dental Sciences, Niigata, Japan (T. Otsuka, M. Uchiyama); Yurin Hospital, Fukushima, Japan (T. Otsuka); The Research Foundation for Microbial Diseases of Osaka University, Kagawa, Japan (Y. Gomi); Biken, Osaka, Japan (Y. Gomi); and National Institute of Infectious Diseases, Tokyo, Japan (N. Inoue)

**Keywords:** varicella, herpes zoster, secondary transmission, Oka vaccine strain, chickenpox, gene 62, viruses, letter

**To the Editor:** Varicella-zoster virus (VZV), a human herpesvirus, is the causative agent of varicella (chickenpox) and herpes zoster (shingles). Worldwide, children are routinely vaccinated with a live attenuated varicella vaccine containing the Oka vaccine (vOka) strain of VZV, originally developed in Japan (*1*–*3*). Although the risk for secondary transmission of the vOka strain from immunocompromised vaccinees to susceptible persons is relatively high, the risk for transmission from immunocompetent vaccinees is low (*1*). We report secondary transmission of the vOka strain from an immunocompetent girl with a history of varicella vaccination to her healthy susceptible brother.

Herpes zoster developed in a healthy 3-year-old girl 2 years after she had received the varicella vaccine (lot VZ040; Biken, Osaka, Japan). She received oral acyclovir treatment and fully recovered by day 19 after herpes zoster onset. On the same day that the girl recovered, her immunocompetent 2-year-old brother was found to have fever and a rash consisting of 10–20 papulovesicles; mild varicella was diagnosed. The boy had no known history of contact with persons infected with varicella or with persons who administered the varicella vaccine. After receiving oral acyclovir treatment, the boy recovered without systemic complications.

On day 19 after the girl’s onset of herpes zoster, an enzyme immunoassay (Denka Seiken, Tokyo, Japan) confirmed the presence of VZV-specific immunoglobulin (Ig) G (titer 48.9, well above the detection limit of 2.0) but not IgM. The boy showed seroconversion of VZV-specific IgG from a titer of <2.0 on day 3 after his disease onset to 19.3 on day 30. Although vesicular fluid or crust specimens were obtained from both children, only the specimens from the boy contained detectable amounts of VZV DNA.

To determine whether vOka or a wild-type VZV strain caused the varicella in this boy, we performed PCR to amplify the entire region of gene 62 and determine its sequence, as described previously (*4*). The DNA sequence of the PCR product matched that of the vOka sequence with the exception of a single wild-type nucleotide substitution at position 105705 (Figure, GenBank accession no. AB497598). Restriction fragment length polymorphism (RFLP) analysis of the PCR products of the open reading frame (ORF) 38 and ORF54 loci using *Pst*I and *BgII* (*5*) demonstrated that the strain had a vOka-like pattern, i.e., *Pst*I-*BgII* +. Furthermore, the vOka-specific sequences at positions 5,745 and 94,167 were conserved in the strain. Taken together, these results indicate that the strain in the boy likely was derived from the vaccine but was not a recombinant between the Biken vOka strain and a wild-type virus.

**Figure:**
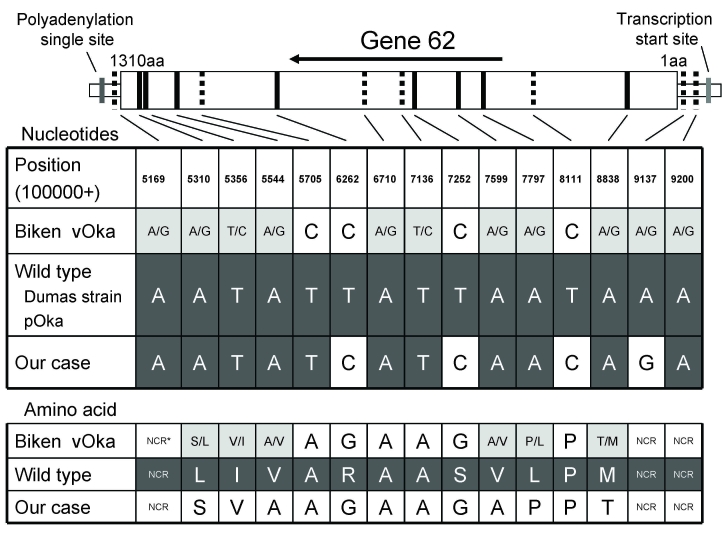
Sequence of gene 62 from patient with varicella from secondary transmission of Oka vaccine strain (vOka). The diagram at the top shows the structure of gene 62. Amino acid residues are numbered 1–1310 from the amino terminus to the carboxyl terminus. Vertical lines indicate the positions of 15-nt base differences between vOka (GenBank accession no. AB097932) and parental (pOka, accession no. AB097933) strains. The 15 **boldface** and broken lines show substitutions with and without amino acid (aa) alterations, respectively. The charts show a comparison of the gene 62 sequences among vOka, pOka, Dumas (accession no. X04370), and the strain isolated from the secondary case-patient (accession no. AB497598). The black, white, and gray boxes denote pOka-type substitutions, vOka-type substitutions, and mixed-type substitutions (mixture of pOka and vOka nucleotides), respectively. NCR, noncoding region.

Commercial varicella vaccines produced by major manufacturers such as Biken, Merck (Rahway, NJ, USA), and GlaxoSmithKline Biologicals (Rixensart, Belgium) possess similar immunogenicity and safety characteristics (*2*,*3*,*6*). Adverse events involving the vOka products from Merck (e.g., rash, varicella, herpes zoster, neurologic complications, and secondary transmission) have been reported at an overall rate of 3.4–5.3 events/10,000 doses given in the United States (*2*,*6*). Six cases of secondary transmission from 5 immunocompetent persons who had received vOka made by Merck have been documented (*2*,*7*,*8*). Unfortunately, the association of vOka from Merck with some of those cases was defined by RFLP analyses of only 1 or 2 loci (*2*,*7*). Although 5 of the 6 cases of secondary transmission were linked with vOka-associated cases of varicella, 1 was transmitted from a vaccinee with herpes zoster (*7*). The fact that the sibling reported in that case was already vaccinated before varicella developed confounds the case.

Postmarketing surveillance conducted in Japan by Biken and the governmental Relief Systems for Adverse Reactions have identified no cases of secondary transmission since Biken’s vOka was licensed in 1985 (Y.G. and N.I., unpub. data). Thus, the case reported here is considered to be rare in that vOka was transmitted to a healthy susceptible person through close contact with a vaccinee with herpes zoster.

vOka is composed of a mixture of genotypically distinct virus strains that have 15 base substitutions in gene 62 compared with the parental Oka strain. Gomi et al. have suggested that the amino acid alterations in the gene 62 products of vOka are associated with the characteristics of vOka, i.e., slower growth and less efficient cell-to-cell spreading in vitro compared with parental Oka (*4*). Previous studies have suggested that some alleles (positions 107797, 105169, 105356, and 108838) in gene 62 were implicated in the formation of vaccine-associated rash (*9*,*10*). The virus in the case-patient reported here contained 1 synonymous nucleotide substitution from Biken’s vOka to a wild-type at position 105705 in gene 62. Because no such alteration was detected in the final Biken vOka products, information about the in vivo process of natural selection for the particular genetic profile is needed.
